# Author Correction: NeoR, a near-infrared absorbing rhodopsin

**DOI:** 10.1038/s41467-024-48357-3

**Published:** 2024-05-10

**Authors:** Matthias Broser, Anika Spreen, Patrick E. Konold, Enrico Schiewer, Suliman Adam, Veniamin Borin, Igor Schapiro, Reinhard Seifert, John T. M. Kennis, Yinth Andrea Bernal Sierra, Peter Hegemann

**Affiliations:** 1 Institute for Biology, Experimental Biophysics, Humboldt-Universität zu Berlin, 10115 Berlin, Germany; 2 Department of Physics and Astronomy, Faculty of Science, Vrije Universiteit Amsterdam, De Boelelaan 1081, 1081 HV Amsterdam, The Netherlands; 3 Fritz Haber Center for Molecular Dynamics, Institute of Chemistry, The Hebrew University of Jerusalem, 9190401 Jerusalem, Israel; 4 Molecular Sensory Systems, Center of Advanced European Studies and Research (caesar), Ludwig-Erhard-Allee 2, 53175 Bonn, Germany

**Keywords:** Biochemistry, Biophysics, Computational biology and bioinformatics

Correction to: *Nature Communications* 10.1038/s41467-020-19375-8, published online 10 November 2020

Since the publication of this work, Enrico Schiewer has changed their name from Enrico Peter. This has now been amended in the HTML and PDF versions of the article.

