# Supplementary material for: NeoR, a near-infrared absorbing rhodopsin
Source: Nat Commun. 2020 Nov 10;11:5682. doi: 10.1038/s41467-020-19375-8 (PMC7655827; doi:10.1038/s41467-020-19375-8)
Supplement: Supplementary file 1 — Supplementary Information [file 41467_2020_19375_MOESM1_ESM.pdf]

Supplementary Materials for

NeoR, a near-infrared absorbing rhodopsin

Matthias Broser[1]*, Anika Spreen[1], Patrick E. Konold[2], Enrico Schiewer[1], Suliman Adam[3], Veniamin Borin[3], Igor Schapiro[3], Reinhard Seifert[4], John T.M. Kennis[2], Yinth Andrea Bernal Sierra[1], and Peter Hegemann[1]

[1]Institute for Biology, Experimental Biophysics, Humboldt-Universität zu Berlin, 10115 Berlin, Germany

[2]Department of Physics and Astronomy, Vrije Universiteit Amsterdam, De Boelelaan 1081, 1081 HV Amsterdam, The Netherlands

[3]Fritz Haber Center for Molecular Dynamics, Institute of Chemistry, The Hebrew University of Jerusalem, Jerusalem 9190401, Israel

[4]Molecular Sensory Systems, Center of Advanced European Studies and Research (caesar), Ludwig-Erhard-Allee 2, 53175, Bonn, Germany

*Correspondence to: matthias.broser@hu-berlin.de

- **Supplementary Figs 1 to 12**
- **Supplementary Table 1**
- **Supplementary Methods**
- **Supplementary References**
- **Supplementary Data 1 (table of microbial rhodopsin sequences)**
- **Supplementary Data 2 (list of primers)**

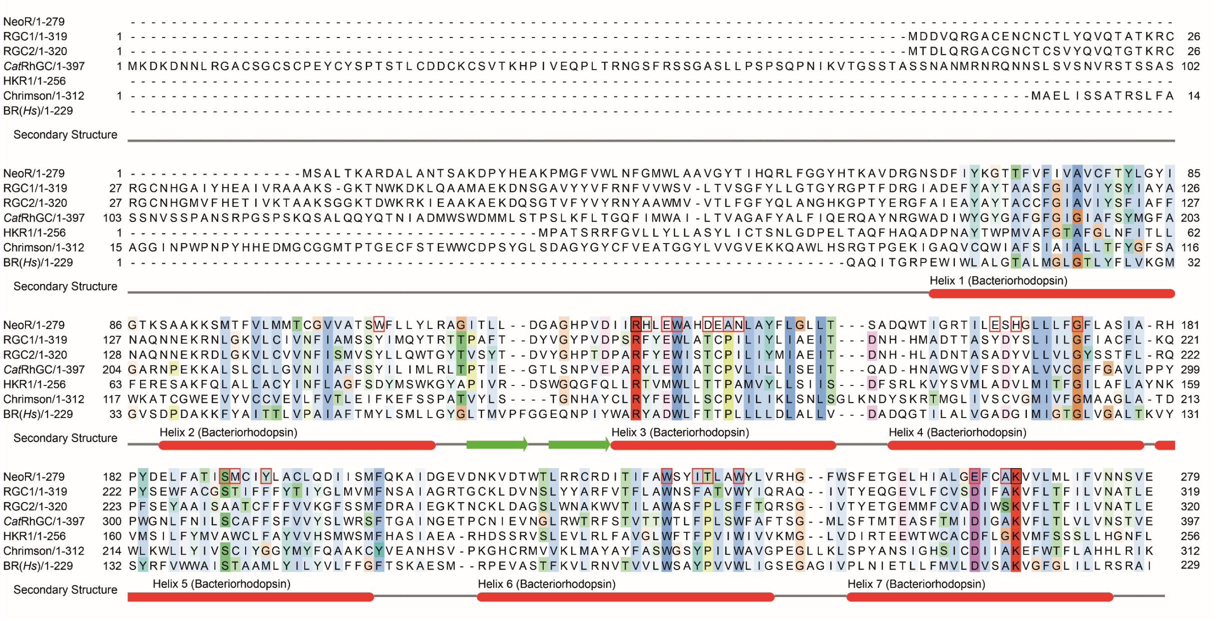

Fig. 1. Sequence alignment of the NeoR rhodopsin domain and homologs. A selection of microbial rhodopsin domain homologs from *Rhizoclosmatium globosum* (NeoR, UniProtKB A0A1Y2CSJ0; RGC1, UniProtKB A0A1Y2CSF8; and RGC2, UniProtKB A0A1Y2CSL9), *Catenaria anguillulae* (*Cat*RhGC, gb MF939579), *Chlamydomonas reinhardtii* (HKR1, gb 410699688), *Chlamydomonas noctigama* (Chrimson, gb KF992060), and *Halobacterium salinarum* (BR(*Hs*), gb 10580964) were aligned using ClustalO [1]. Amino acid residues are colored according to their extent of conservation, with a 30% filter, and residues mutated in this study are framed. Secondary structure elements are derived from the crystal structure of bacteriorhodopsin (BR; PDB ID: 2i21) [2].

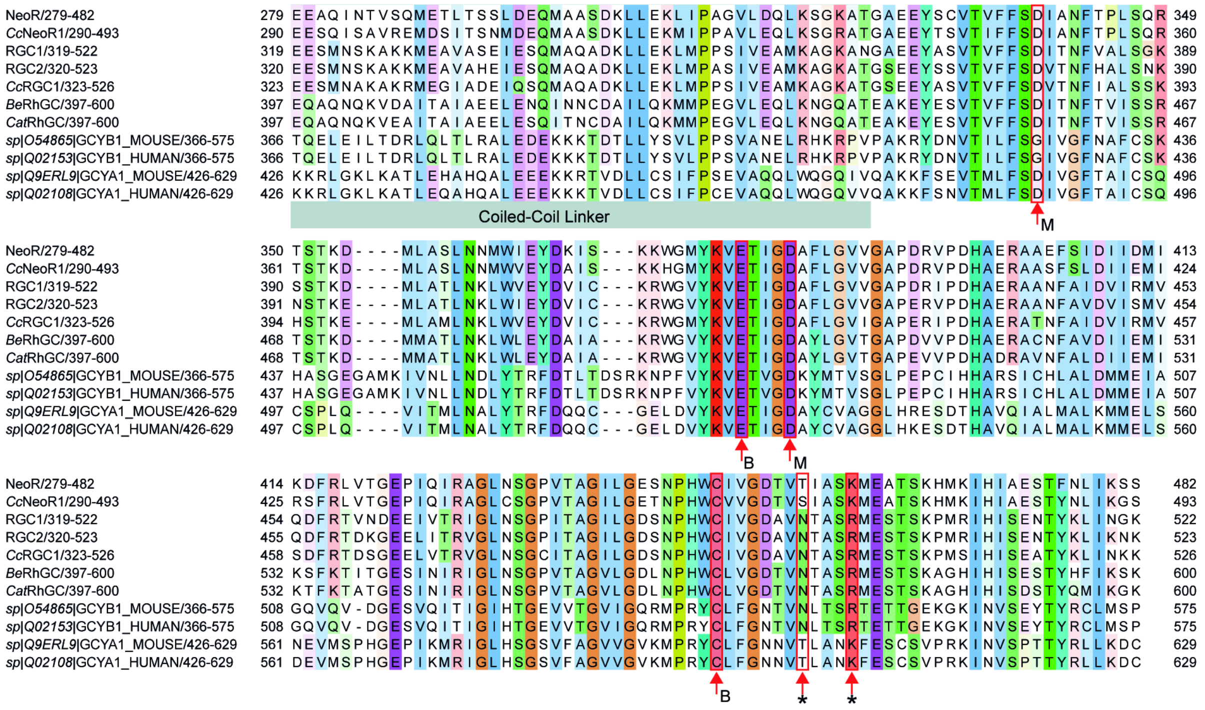

Fig. 2. Sequence alignment of the NeoR linker, the cyclase domain and homologs. A selection of homolog guanylyl-cyclases: RGCs from *Rhizoclosmatium globosum* (NeoR, UniProtKB A0A1Y2CSJ0; RGC1, UniProtKB A0A1Y2CSF8; and RGC2, UniProtKB A0A1Y2CSL9), closely related *Chytridium confervae* (*Cc*NeoR1 UniProtKB A0A507FHL0; *Cc*RGC1 UniProt A0A507F303), *Blastocladiella emersonii* (BeRhGC gb AIC07007.1), *Catenaria anguillulae* (CatRhGC gb MF939579), soluble guanylyl cyclase from mouse (beta1 subunit (sCG beta1(mouse) UniprotKB O54865); alpha1 subunit (sGC alpha1(mouse) UniprotKB Q9ERL9) and human soluble guanylyl cyclase (beta1 subunit (sCG beta1(human) UniprotKB Q02153); alpha1 subunit (sGC alpha1(human) UniprotKB Q02108). Amino acid residues are colored according to their extent of conservation, with a 30% filter, and selected catalytic relevant residues are depicted: B: base discriminating residues; M: metal-binding residues; *: transition-state stabilizing residues. NeoR and RGC1/2 linker region and cyclase domain are highly conserved: linker region (46 amino acids): 43%/59% identity/similarity between NeoR and RGC1/2 and 96%/96% between RGC1 and RGC2; cyclase domain (185 amino acids): 65%/77% identity/similarity for NeoR and RGC1/2 and 88%/93% between RGC1 and RGC2. Notably, NeoR and ccNeoR1 are missing two catalytically relevant amino acids discussed to stabilize the transition state (marked with *), resembling the situation in the alpha-subunit of vertebrate soluble guanylyl cyclase that functions as obligate heterodimer.

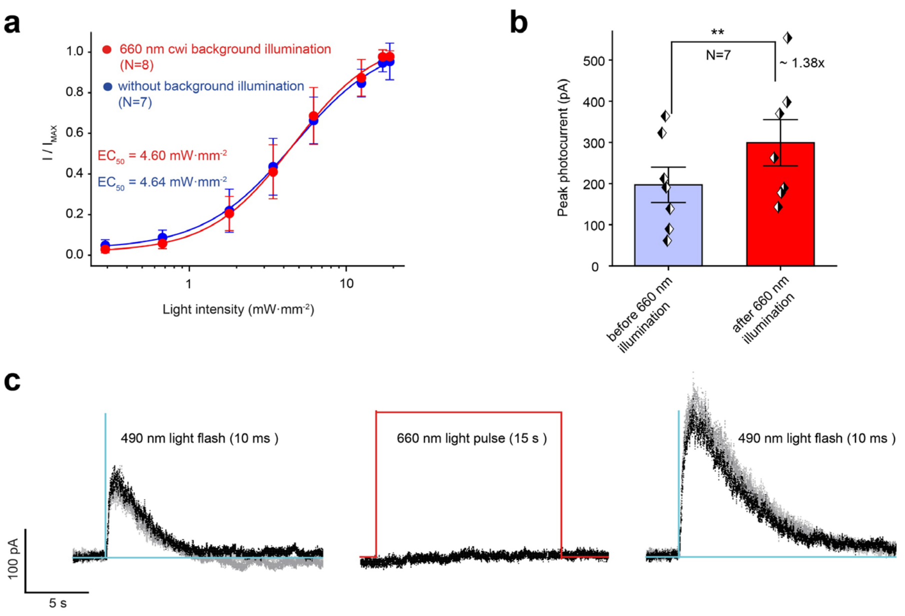

Fig. 3. Patch clamp measurements of RGC2/NeoR complexes in ND7/23 cells upon far-red illumination. (a) Normalized light titration curves (means ± s.d., N = number of biological replicates) with (red cycles) and without (blue cycles) 660 nm continues wave (background) illumination (cwi). The same data as used for main Fig. 1e, but normalization to I_{MAX} within each cell, cancelled out the high cell to cell variance. **(b)** Peak photocurrents amplitudes before (purple) and after (red) 15s illumination of the cell (mean ± s.e.m., N = number of biological replicates) Photocurrent was evoked by 10 ms 490 nm light flashes and datapoints represent the average of three sequentially recorded sweeps. Significance of the photocurrent increase was proven by a one-sided, paired stundent´s t-test with p = 0.00538 (marked with **) and an effect size of ~1.38. **(c)** Representative photocurrent traces before (left panel) during (middle panel) and after (right panel) illumination of a cell for 15 s with 660 nm light. Current traces recorded during or immediately before and after far-red illumination are shown in black, additional recorded sweeps are drawn in gray.

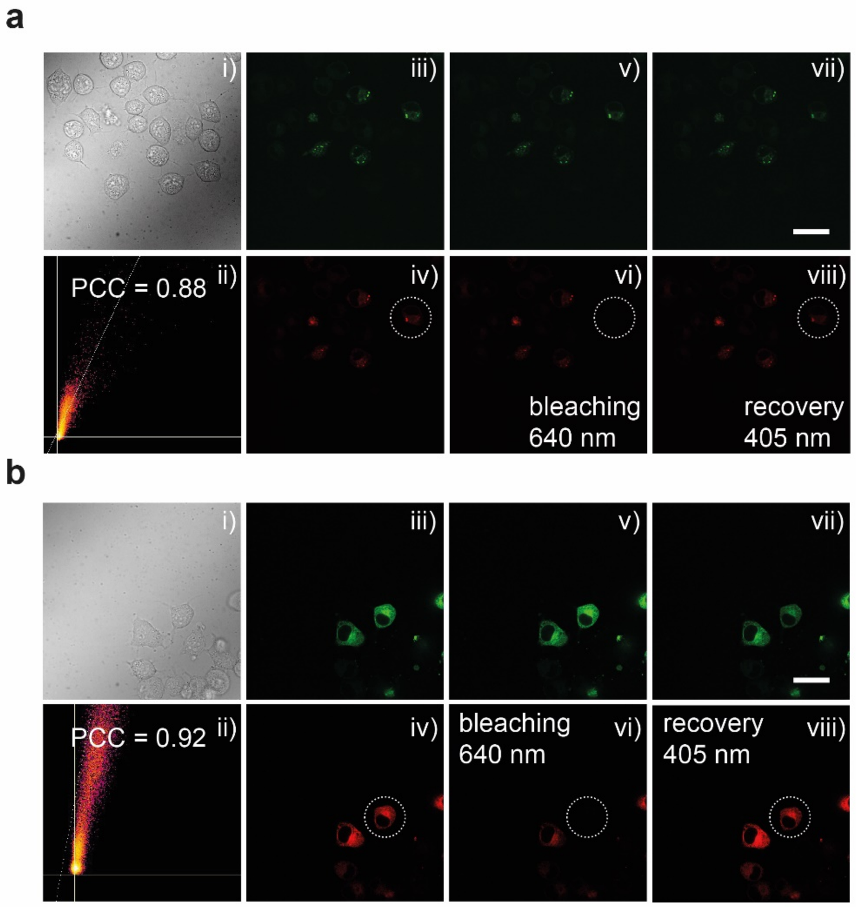

Fig. 4. Confocal Images of RGC1-YFP/NeoR and RGC2-YFP/NeoR complexes in ND7/23 cells. **(a)** RGC1/NeoR complexes in ND7/23 cells: i) DIC Image of transfected cells. ii) scatter plot of joint relationships between RGC1-YFP and (intrinsic) NeoR fluorescence calculated within the region of interest (ROI: see cycle in iv) from images iii) and iv); PCC: Pearson's correlation coefficient. iii) RGC-YFP fluorescence (green) and iv) NeoR fluorescence (red) before bleaching of the ROI (cycle in iv)). v) + vi) RGC-YFP fluorescence (green) and NeoR fluorescence (red) after bleaching of the ROI with 640 nm light. vii) + viii) RGC-YFP fluorescence (green) and NeoR fluorescence (red) after recovery of NeoR fluorescence by illumination with 405 nm light (scale bar, 100 µm). **(b)** RGC2/NeoR complexes in ND7/23 cells: images i)-viii) analog to **(a)**. The reversible bleaching of NeoR fluorescence in single cells was repeated in 2 independent experiments using 5 fields of view each.

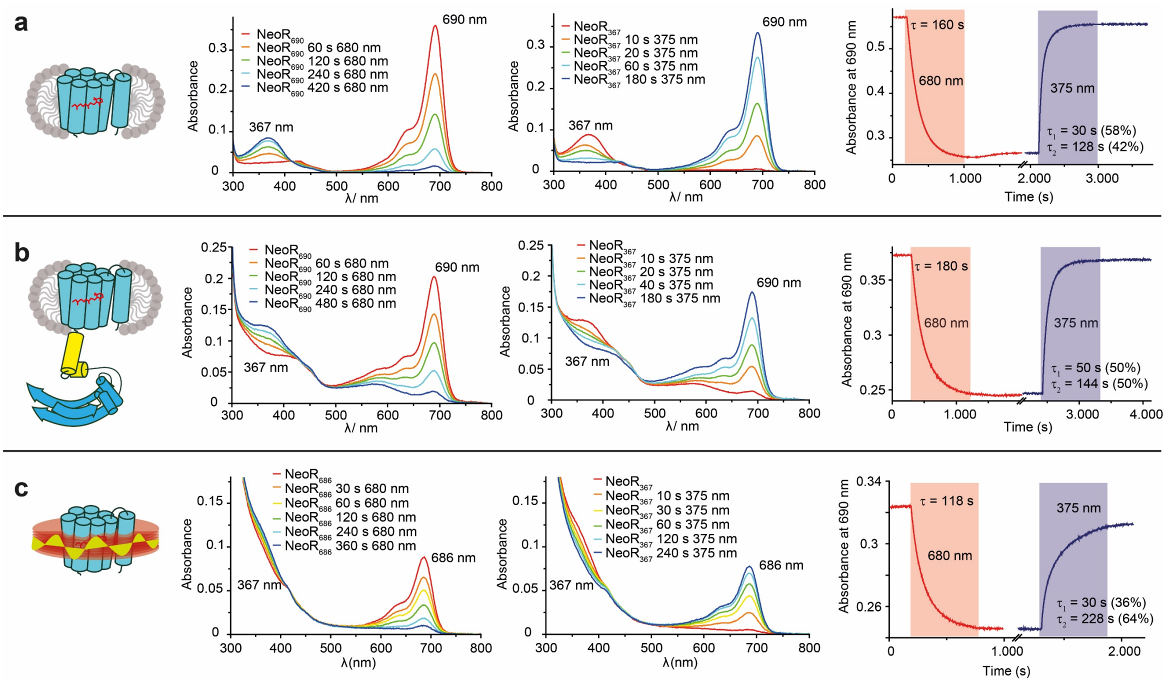

Fig. 5. Comparative overview of the spectral features from different NeoR protein samples. (a) NeoR rhodopsin fragment in detergent micelles (also shown in Fig. 2 of the main text). **(b)** NeoR full length protein in detergent micelles and **(c)** NeoR rhodopsin fragment in styrene-maleic acid copolymer (SMA 3:1) lipidic nanodiscs. Kinetic traces of 680 nm photobleaching (red box) were fitted by a single exponential, recovery traces by illumination with 375 nm (purple box) were fitted with two exponentials; lifetimes and relative amplitudes are indicated.

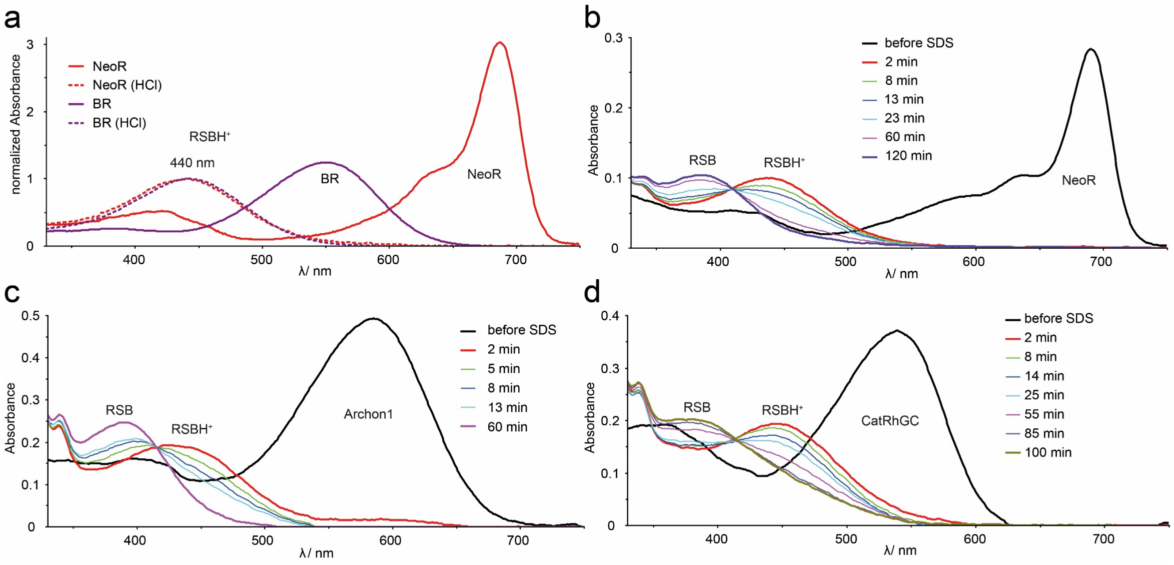

Fig. 6. Detergent and acid denaturation of NeoR. (a) Spectra of NeoR and bacteriorhodopsin (BR) before and after acid denaturation in 100 mM HCl. Spectra are normalized according to the 440 nm absorbance of the $RSBH^+$. Based on an extinction coefficient of ε440 = 43,000 $M^{-1}cm^{-1}$ for all-trans retinal [3], we calculated ε690 = 129,000 $M^{-1}cm^{-1}$ for NeoR. **(b)** The purified rhodopsin domains of NeoR, **(c)** Archon1 and **(d)** CatRhGC were denatured with 2% sodium dodecyl sulfate (SDS), and absorption spectra were recorded at the indicated time points after SDS treatment.

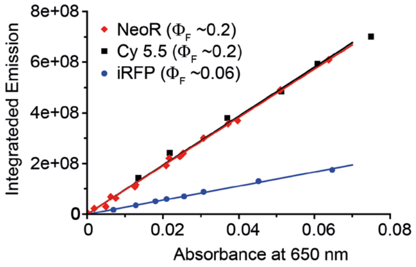

Fig. 7. Determination of NeoR$_{690}$ fluorescence quantum yield. The NeoR fluorescence quantum efficiency was determined to be ΦF = 0.2, based on comparison with iRFP [4] and the organic dye Cy 5.5. Linear fit (lines) of the integrated fluorescence (filled symbols) plotted over the absorbance at the excitation wavelength (650 nm).

a)

#	Protonation			λ_{max}	
	E136	D140	E262	nm	eV
1				378	3.28
2	x			449	2.76
3		x		466	2.66
4			x	469	2.65
5	x	x		599	2.07
6	x		x	611	2.03
7		x	x	612	2.02
8	x	x	x	696	1.78
BR				496	2.50
KR2				475	2.61

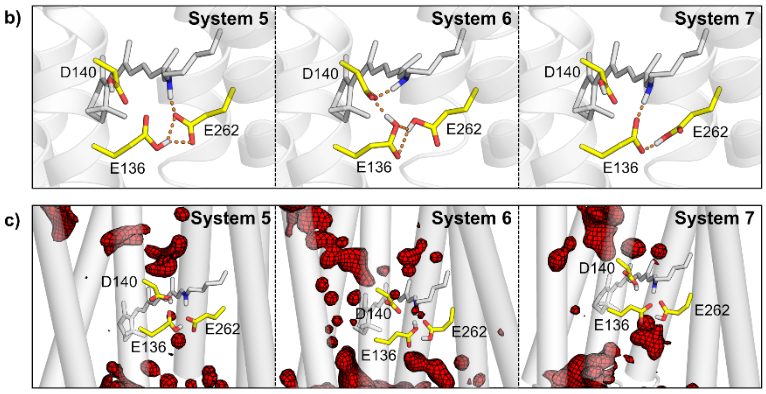

Fig. 8. Alternate QM/MM optimerization of NeoR based on a reduced QM region, including the side chain of K266 and the RSB. (a) Overview of QM/MM excitation energy calculations of the NeoR active site with varying protonation of E136, D140 and E262 as indicated. λ_{MAX} refers to the absorption maxima as derived from excited-state energy calculation. Applying the alternate approach with only the RSB and K266 in the QM region to bacteriorhodopsin (BR) and Krokinobacter eikastus rhodopsin 2 (KR2) yielded a λ_{MAX} of 496 nm (2.50 eV) and 475 nm (2.61 eV), respectively. **(b)** Active site from QM/MM-optimized models, with hydrogen bonds represented as dashed lines. **(c)** Water intrusion into NeoR as observed during subsequent MD simulations with water densities visualized as red meshes.

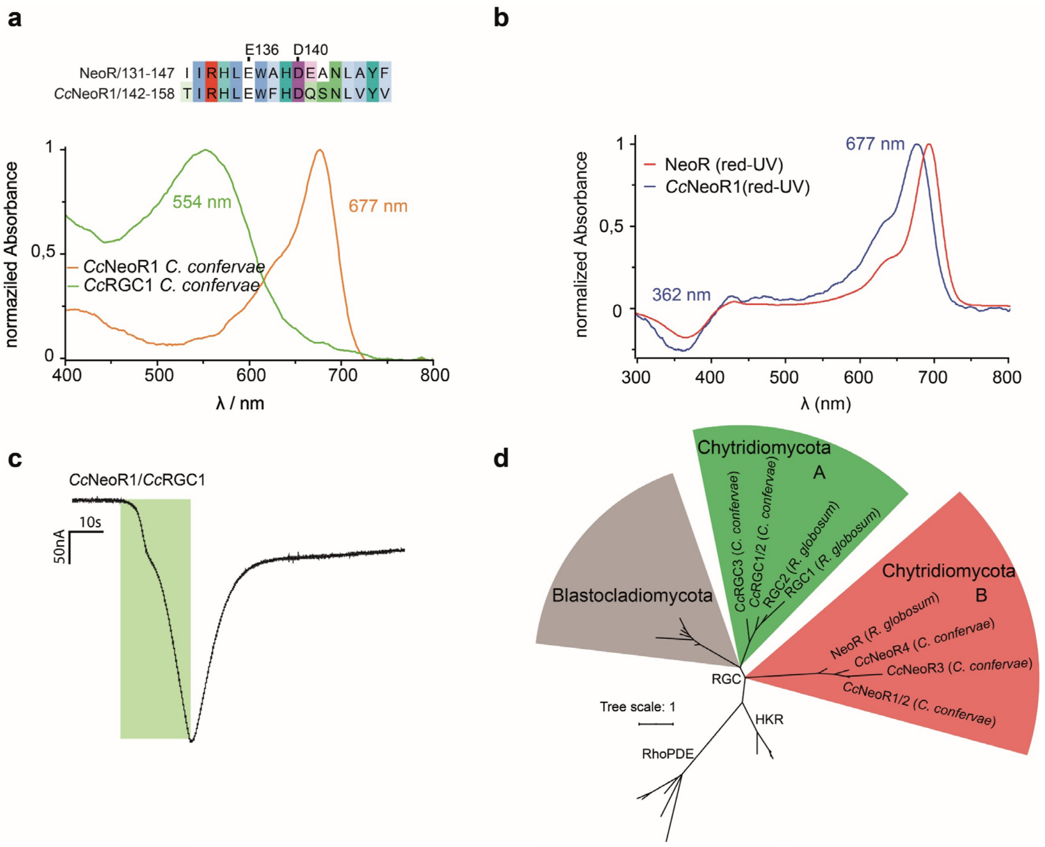

Fig. 9. NeoR/RGC from *C. confervae* and phylogenetic tree of enzymerhodopsins. (a) Normalized UV-vis spectra from *C. confervae Cc*NeoR1 and *Cc*RGC1 (Uniprot: A0A507FHL0 and A0A507F303, respectively) purified from human embryonic kidney (HEK-T) cells. Conserved sequences from canonical transmembrane helix 3 of NeoR (*R. globosum*) and *Cc*NeoR1 (*C. confervae*), with important RSBH$^+$ residues E136 and D140 highlighted. **(b)** Normalized difference spectrum of *Cc*NeoR1 after bleaching with far-red light (680 nm) with similar spectrum obtained for NeoR. **(c)** Two-electrode voltage clamp measurement of *Cc*NeoR1 coexpressed with *Cc*RGC1 and a cGMP-sensitive reporter channel in *xenopus* oocytes. Photocurrents have been evoked by applying a 20 s light pulse (green box). **(d)** Unrooted phylogenetic tree derived from the rhodopsin domains of selected enzymerhodopsins; scale bar represents average number of amino acid substitutions per site. RGC branches are highlighted: Blastocladiomycota (black/grey), Chytridiomycota branch A. (green; includes RGC1 [*R. globosum*; UniProt A0A1Y2CSF8], RGC2 *[R. globosum*; UniProt A0A1Y2CSL9], *Cc*RGC1 [*C. confervae*; UniProt A0A507F303], *Cc*RGC2 [*C. confervae*; UniProt A0A507FES2], and *Cc*RGB3 [*C. confervae*; UniProt A0A507F303]), and Chytridiomycota branch B. (red; includes NeoR [*R. globosum*; UniProt A0A1Y2CSJ0], *Cc*NeoR1 [*C. confervae*; UniProt A0A507FHL0], *Cc*NeoR2 [*C. confervae*; UniProt A0A507F0Q5], *Cc*NeoR3 [*C. confervae*; UniProt A0A507FPT7], and *Cc*NeoR4 [*C. confervae*; UniProt A0A507F3A5]).

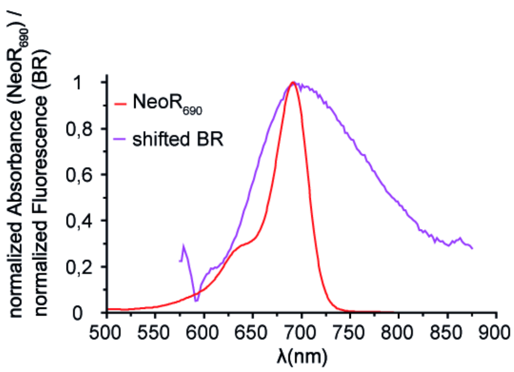

Fig. 10. Spectral overlap of RGC1 fluorescence (magenta) and NeoR absorption (red). The RGC1 fluorescence was approached by bacteriorhodopsin fluorescence[5] blue-shifted by 25 nm.

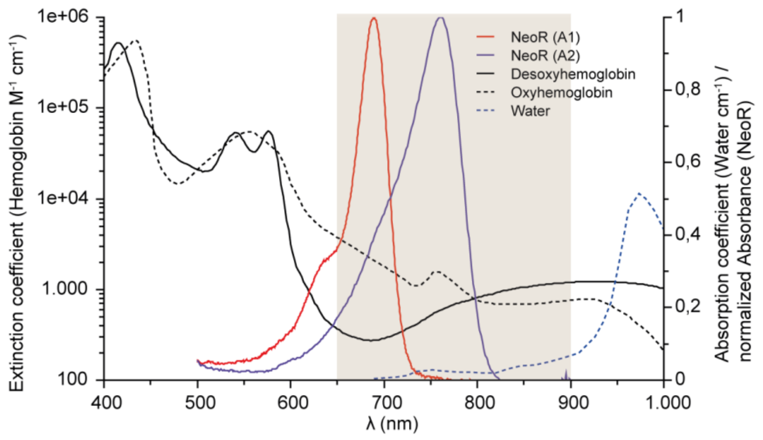

Fig. 11. Spectral comparison of NeoR with the NIR window of biological tissue (650–900 nm) derived from the absorption of hemoglobin, deoxyhemoglobin, and water. Spectra were obtained from S. Prahl, 1998 (scott.prahl@oit.edu), using data from W.B. Gratzer, Medical Research Council Labs, Holly Hill, London, UK and N. Kollias, Wellman Laboratories, Harvard Medical School, Boston, MA, USA. https://omlc.org/spectra/hemoglobin/summary.html

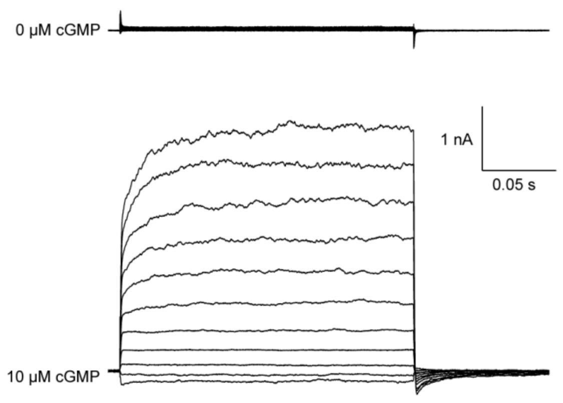

Fig. 12. Current traces of the engineered cGMP-gated SthK channel. Whole-cell currents of an engineered cGMP-gated SthK channel expressed in CHO cells in the absence (upper panel) and presence of cGMP (10 μM, lower panel).

Table S1. Mutants of NeoR, related mutations in BR, and their spectral properties.

NeoR mutations	λ_{max} (nm)	Δ_{max} (nm)	sw	FL-QY (%)	homologous BR mutations	λ_{max} (nm)	Δ_{max} (nm)	Ref. BR
WT	690_d		+	20		$568_p/561_d$		
W110Y	677_d	-13	+		Y57F	548_d	-21	[6]
R133Q	n.C.	--	--		R82N	594_d	+33	[7]
H134Y	686_d	-6	+		Y83F	540_d	-21	[6]
E136D	n.C.	--	--		D85E	546_d	-15	[8]
E136Q	420_d	-270	dp		D85Q	566	-2	[9]
E136C	n.C.	--	--					
W137Y	677_d	-13	+					
W137F	677_d	-13	+					
D140N	686_d	-4	+		T89D	560_d	-1	[10]
D140C	676_d	-14	+	13				
D140T	630_d	-60	+	5				
E141C	697_d	+7	+		T90V	549_d	-12	[10]
E141Q	684_d	-6	+	22				
G173L	n.C.	--	-		G122C	524_d	-37	[11]
S191A	675_d	-16	+	15	S141A	544_d	-17	[10]
M192L	686_d	-4	+		T142V	553	-8	[10]
Y195F	692_d	-2	+		M145A	477	-84	[12]
W234F	693_d	+3	+		W182F	477_d	-84	[6]
I237Y	n.C.	--	-		Y185F	573	+12	[13]
I237A	n.C.	--	-					
T238P	662_d	-28	+		P186A	576	+15	[6] [11]
T238A	664_d	-26	+	10	P186L	459_d	-102	[14]

W241H	687_d	-3	+					
E262Q	394_d	-296	dp		D212N	548_d<br>584_P	-13<br>+23	15<br>16
E262D	n.C.	--	-					
A265S	683_d	-7	+					
A265T	n.C.	--			A215T	550_d	-18	17
K266A rb	n.C.	--			K216 rb	n.C.	--	
W110Y, M192L	675_d	-15	+					
W110Y, A265S	$657/630_d$	-33/-60	+	8				
W110Y, D140C	658_d	-32	+					
D140C, S191A	643_d	-47	+	5				
T238A, S191A	625_d	-65	+*					
D140T, E141C A142P, N143V	631_d	-59	+	4				

Underlined proteins are included in Fig. 3C of the main text; rb, retinal-binding lysine; λ_{max}, peak absorption expressed in nm; n.C., no chromophore; sw, bimodal switchable; dp, RSB permanently deprotonated; FL-QY, fluorescence quantum yield in %; d, measured in detergent; p, measured in purple membranes; Ref. BR, references that describe homologous mutations in bacteriorhodopsin.

Supplementary Methods

Calculation of potential FRET energy transfer rates.

The FRET calculations were based on the following expression[18]

$$k_{DA} = \frac{k^2}{R^6}\frac{k_\Gamma}{n^4} I$$

Where k_{DA} denotes the energy transfer rate in ps^{-1}. Here R denotes the center-to-center distance between donor and acceptor, k^2 the orientation factor of donor and acceptor, k_r the radiative rate of the donor in ns^{-1}, n the refractive index of the medium and I the spectral overlap term:

$$I = 8.8 \cdot 10^{17} \int \frac{\varepsilon_A(v) f_D(v)}{v_4} dv$$

In the spectral overlap expression, expressed in wavenumbers, ε_A denotes the extinction coefficient of the acceptor in M^{-1} cm^{-1}, f_D the fluorescence spectrum of the donor with the integrated fluorescence normalized to 1 and ν the frequency.

For our calculations, we set k_r at $(6\ ns)^{-1}$ [19] and assume that n = 1.6[18]. In the absence of an experimental fluorescence spectrum of RGC1, we took a published spectrum of bacteriorhodopsin[5] and blue-shifted it by 25 nm. Supplementary Fig. S8 shows the spectral overlap between RGC1 and NeoR. In the absence of detailed structural information, we assumed that the orientation factor k^2 = 2/3, which is valid for isotropic orientations. We then calculated k_{DA} for R = 20, 25 and 30 Å, and found k_{DA} = 1.18 , 0.31 and 0.10 ps^{-1}, respectively, which corresponds to FRET lifetimes of 840 fs, 3.2 ps and 10 ps, respectively.

QM/MM simulation of NeoR with a smaller QM region

To study the impact of the QM region size we have computed excitation energies with the QM region composed of retinal PSB and the side chain of K266. By using a smaller QM region, we were able to sample a larger amount of initial conditions for the different protonation states of the counterions. Whenever a counterion was to be protonated, we placed the proton on either of the two carboxylate oxygens, once in *cis* and once in *trans* conformation, thereby creating four different starting geometries for each protonated residue. For models with multiple protonated counterions, we generated all possible combinations of initial proton placements. These initial geometries were then minimized analogously to the large QM region, i.e. with QM/MM using B3LYP/cc-pVDZ and the CHARMM force field. For each protonation state, we chose the lowest energy geometry for the computation of excitation energies with RI-ADC(2).

Calibration of the QM/MM protocol using bacteriorhodopsin (BR) and *Krokinobacter eikastus* rhodopsin 2 (KR2)

To assess the accuracy of the QM/MM protocol, additional simulations were performed for BR and KR2 with the QM region size, as descrived above. The initial geometries for these calculations were obtained from the protein data bank (PDB), ID: 5J7A[20] and 6TK6[21]. Geometry optimization was performed with QM region consisting of retinal along with the side chain of covalently attached lysine residue and treated at B3LYP/cc-pVDZ level of theory. Remaining part of the protein was treated with molecular mechanics from AMBER force-field. These calculations were performed using Chemshell program[22] interfaced with quantum chemistry package Orca[23]. Excitation energies were calculated with RI-ADC(2) method using the Turbomole program[24]. The resulted excitation energies based on the reduced model QM/MM calculations are (514 nm) 2.41 eV and (488 nm) 2.54 eV, for BR and KR2, respectively. For

consistency with the previous simulations, the excitation energy was also computed with CHARMM partial charges for the MM region using the Turbomole program. The excitation energies are found to be in close agreement with the results obtained with the AMBER force field: 496 nm (2.50 eV) for BR and 475 nm (2.61 eV) for KR2 (Fig. S8). Both results are systematically underestimating the experimental absorption maxima, in line with the NeoR results, providing further confidence in the chosen simulation protocol.

Supplementary References

1 Sievers, F. & Higgins, D. G. Clustal omega. *Curr Protoc Bioinformatics* **48**, 3 13 11-16, doi:10.1002/0471250953.bi0313s48 (2014).

2 Lanyi, J. K. & Schobert, B. Propagating structural perturbation inside bacteriorhodopsin: crystal structures of the M state and the D96A and T46V mutants. *Biochemistry* **45**, 12003-12010, doi:10.1021/bi061310i (2006).

3 Barua, A. B., Olsen, J. A., Furr, H. C. & van Breemen, R. B. in *Chromatographics Science SeriesModern Chromatographic analysis of Vitamins* Vol. 84 *Modern Chromatographic Analysis of Vitamins* Ch. 1, (Marcel Dekker, Inc., 2000).

4 Filonov, G. S. *et al.* Bright and stable near-infrared fluorescent protein for in vivo imaging. *Nat Biotechnol* **29**, 757-761, doi:10.1038/nbt.1918 (2011).

5 Kennis, J. T. M. *et al.* Ultrafast protein dynamics of bacteriorhodopsin probed by photon echo and transient absorption spectroscopy. *Journal of Physical Chemistry B* **106**, 6067-6080, doi:10.1021/jp014681b (2002).

6 Mogi, T., Marti, T. & Khorana, H. G. Structure-function studies on bacteriorhodopsin. IX. Substitutions of tryptophan residues affect protein-retinal interactions in bacteriorhodopsin. *J Biol Chem* **264**, 14197-14201 (1989).

7 Miercke, L. J. *et al.* Wild-type and mutant bacteriorhodopsins D85N, D96N, and R82Q: purification to homogeneity, pH dependence of pumping, and electron diffraction. *Biochemistry* **30**, 3088-3098, doi:10.1021/bi00226a016 (1991).

8 Greenhalgh, D. A. *et al.* Effect of introducing different carboxylate-containing side chains at position 85 on chromophore formation and proton transport in bacteriorhodopsin. *J Biol Chem* **267**, 25734-25738 (1992).

9 Saeedi, P., Moosaabadi, J. M., Sebtahmadi, S. S., Behmanesh, M. & Mehrabadi, J. F. Site-directed mutagenesis in bacteriorhodopsin mutants and their characterization for bioelectrical and biotechnological equipment. *Biotechnol Lett* **34**, 455-462, doi:10.1007/s10529-011-0731-4 (2012).

10 Marti, T. *et al.* Bacteriorhodopsin mutants containing single substitutions of serine or threonine residues are all active in proton translocation. *J Biol Chem* **266**, 6919-6927 (1991).

11 Ahl, P. L. *et al.* Effects of amino acid substitutions in the F helix of bacteriorhodopsin. Low temperature ultraviolet/visible difference spectroscopy. *J Biol Chem* **263**, 13594-13601 (1988).

12 Greenhalgh, D. A., Farrens, D. L., Subramaniam, S. & Khorana, H. G. Hydrophobic amino acids in the retinal-binding pocket of bacteriorhodopsin. *J Biol Chem* **268**, 20305-20311 (1993).

13 Mogi, T., Stern, L. J., Hackett, N. R. & Khorana, H. G. Bacteriorhodopsin mutants containing single tyrosine to phenylalanine substitutions are all active in proton translocation. *Proc Natl Acad Sci U S A* **84**, 5595-5599, doi:10.1073/pnas.84.16.5595 (1987).

14 Inoue, K. *et al.* Red-shifting mutation of light-driven sodium-pump rhodopsin. *Nat Commun* **10**, 1993, doi:10.1038/s41467-019-10000-x (2019).

15 Mogi, T., Stern, L. J., Marti, T., Chao, B. H. & Khorana, H. G. Structure-Function Studies on Bacteriorhodopsin .7. Aspartic-Acid Substitutions Affect Proton Translocation by

Bacteriorhodopsin. *Proceedings of the National Academy of Sciences of the United States of America* **85**, 4148-4152, doi:Doi 10.1073/Pnas.85.12.4148 (1988).
16 Cao, Y. *et al.* Water is required for proton transfer from aspartate-96 to the bacteriorhodopsin Schiff base. *Biochemistry* **30**, 10972-10979 (1991).
17 Spudich, E. N. *et al.* A Transporter Converted into a Sensor, a Phototaxis Signaling Mutant of Bacteriorhodopsin at 3.0 Å. *Journal of Molecular Biology* **415**, 455-463, doi:https://doi.org/10.1016/j.jmb.2011.11.025 (2012).
18 Kleima, F. J. *et al.* Forster excitation energy transfer in peridinin-chlorophyll-a-protein. *Biophysical journal* **78**, 344-353, doi:10.1016/s0006-3495(00)76597-0 (2000).
19 Lenz, M. O. *et al.* First steps of retinal photoisomerization in proteorhodopsin. *Biophysical journal* **91**, 255-262, doi:10.1529/biophysj.105.074690 (2006).
20 Nogly, P. *et al.* Lipidic cubic phase injector is a viable crystal delivery system for time-resolved serial crystallography. *Nature Communications* **7**, 12314, doi:10.1038/ncomms12314 (2016).
21 Skopintsev, P. *et al.* Femtosecond-to-millisecond structural changes in a light-driven sodium pump. *Nature* **583**, 314-318, doi:10.1038/s41586-020-2307-8 (2020).
22 Metz, S., Kästner, J., Sokol, A. A., Keal, T. W. & Sherwood, P. ChemShell—a modular software package for QM/MM simulations. *WIREs Computational Molecular Science* **4**, 101-110, doi:10.1002/wcms.1163 (2014).
23 Neese, F. Software update: the ORCA program system, version 4.0. *WIREs Computational Molecular Science* **8**, e1327, doi:10.1002/wcms.1327 (2018).
24 Balasubramani, S. G. *et al.* TURBOMOLE: Modular program suite for ab initio quantum-chemical and condensed-matter simulations. *The Journal of Chemical Physics* **152**, 184107, doi:10.1063/5.0004635 (2020).
