# Supplementary material for: NeoR, a near-infrared absorbing rhodopsin
Source: Nat Commun. 2020 Nov 10;11:5682. doi: 10.1038/s41467-020-19375-8 (PMC7655827; doi:10.1038/s41467-020-19375-8)
Supplement: Supplementary file 5 — Reporting Summary [file 41467_2020_19375_MOESM5_ESM.pdf]

nature research

Corresponding author(s): Matthias Broser

Last updated by author(s): Oct 2, 2020

Reporting Summary

Nature Research wishes to improve the reproducibility of the work that we publish. This form provides structure for consistency and transparency in reporting. For further information on Nature Research policies, see our Editorial Policies and the Editorial Policy Checklist.

Statistics

For all statistical analyses, confirm that the following items are present in the figure legend, table legend, main text, or Methods section.

n/a	Confirmed	
☐	☒	The exact sample size (*n*) for each experimental group/condition, given as a discrete number and unit of measurement
☐	☒	A statement on whether measurements were taken from distinct samples or whether the same sample was measured repeatedly
☐	☒	The statistical test(s) used AND whether they are one- or two-sided<br>*Only common tests should be described solely by name; describe more complex techniques in the Methods section.*
☒	☐	A description of all covariates tested
☒	☐	A description of any assumptions or corrections, such as tests of normality and adjustment for multiple comparisons
☐	☒	A full description of the statistical parameters including central tendency (e.g. means) or other basic estimates (e.g. regression coefficient) AND variation (e.g. standard deviation) or associated estimates of uncertainty (e.g. confidence intervals)
☐	☒	For null hypothesis testing, the test statistic (e.g. *F*, *t*, *r*) with confidence intervals, effect sizes, degrees of freedom and *P* value noted<br>*Give P values as exact values whenever suitable.*
☒	☐	For Bayesian analysis, information on the choice of priors and Markov chain Monte Carlo settings
☒	☐	For hierarchical and complex designs, identification of the appropriate level for tests and full reporting of outcomes
☒	☐	Estimates of effect sizes (e.g. Cohen's *d*, Pearson's *r*), indicating how they were calculated

Our web collection on statistics for biologists contains articles on many of the points above.

Software and code

Policy information about availability of computer code

Data collection	pCLAMP version 9.0 and 10.4 (Molecular Devices, San Jose, CA, USA), UVProbe v2.34 (Shimadzu Corporation, Kyoto, Japan), FluorEssence™ 2.5.2 (HORIBA Instruments Inc., NJ, USA)
Data analysis	Origin 2017 (Originlab, Northampton, MA), Clampfit 10.7 (Molecular Devices), ClustalO (1.2.4_1), TrimAl (1.4.1), PhyML (3.1_1), iTOL (5.6.3), Glotaran 1.5.1. (Snellenburg et al. 2012), MODELLER 9.20 (https://salilab.org), Dowser 0.26 (https://sourceforge.net/projects/dowser/files/), VMD 1.9.3 (https://www.ks.uiuc.edu), Amber16 (https://ambermd.org), TURBOMOLE V7.3 (http://www.turbomole.com), ChemShell 3.7.0 (https://www.chemshell.org)

For manuscripts utilizing custom algorithms or software that are central to the research but not yet described in published literature, software must be made available to editors and reviewers. We strongly encourage code deposition in a community repository (e.g. GitHub). See the Nature Research guidelines for submitting code & software for further information.

Data

Policy information about availability of data

All manuscripts must include a data availability statement. This statement should provide the following information, where applicable:

- Accession codes, unique identifiers, or web links for publicly available datasets
- A list of figures that have associated raw data
- A description of any restrictions on data availability

Data supporting the findings of this manuscript are available from the corresponding author upon reasonable request. All original data are stored on local hard drives (with backups), and selected plasmids are available from AddGene. Crystal structures used in this study are deposited at the RCSB-Protein Data Bank (https://www.rcsb.org) under PDB ID: 4QI1, 5ZIM, 6GUX, 5JJE, 5AX0, 3WQJ, 4JQ6, 4HYJ, 5ZIH, 3UG9, 6RF6, 6NWD, 6GYH, 2I21.

Field-specific reporting

Please select the one below that is the best fit for your research. If you are not sure, read the appropriate sections before making your selection.

☒ Life sciences ☐ Behavioural & social sciences ☐ Ecological, evolutionary & environmental sciences

For a reference copy of the document with all sections, see nature.com/documents/nr-reporting-summary-flat.pdf

Life sciences study design

All studies must disclose on these points even when the disclosure is negative.

Sample size	All experiments were performed with between 3 to 7 cells to ensure reproducability. No statistic tests were used to predetermine sample size.
Data exclusions	We exclusivly analyzed electrophysiological data from ND7/23 cell recordings with access resistance <10 MOhm and membrane resistance >500 MOhm as written in the Methods section. The data exclusions criteria were established prior to data collection.
Replication	Repeated electrophysiological recordings were performed with at least 7 biological replicants (except the action-spectrum Fig. 1d with N=3) arising from at least 4 different transfection-batches recorded within approx. 6 weeks. In single cell experiments, 1 out of 7 replicants did not support the statistical trend observed upon red-light treatment.<br>For UV-vis and Fluorescence spectral on NeoR Mutants recordings have been recorded on purified protein from single expression batches. For NeoR wildtyp spectra purified protein from at least 3 different expression batches have been used - all reporting the same result.
Randomization	Randomization was not used.
Blinding	Blinding was not performed to ensure correct assignment of the data to the measured constructs and/or experimental conditions.

Reporting for specific materials, systems and methods

We require information from authors about some types of materials, experimental systems and methods used in many studies. Here, indicate whether each material, system or method listed is relevant to your study. If you are not sure if a list item applies to your research, read the appropriate section before selecting a response.

Materials & experimental systems

n/a	Involved in the study	
☒	☐	Antibodies
☐	☒	Eukaryotic cell lines
☒	☐	Palaeontology and archaeology
☐	☒	Animals and other organisms
☒	☐	Human research participants
☒	☐	Clinical data
☒	☐	Dual use research of concern

Methods

n/a	Involved in the study	
☒	☐	ChIP-seq
☒	☐	Flow cytometry
☒	☐	MRI-based neuroimaging

Eukaryotic cell lines

Policy information about cell lines

Cell line source(s)	HEK-T (ECACC 12022001); ND7/23 (ECACC 92090903); Gibco" Sf21 cells (B82101, ThermoFisherScientific)
Authentication	None of the cell lines used were authenticated. Cells were used from the vendors, without further characterization.
Mycoplasma contamination	not tested.
Commonly misidentified lines (See ICLAC register)	No commonly misidentified cell lines were used in the study.

Animals and other organisms

Policy information about studies involving animals; ARRIVE guidelines recommended for reporting animal research

Laboratory animals	Mature Xenopus laevis, female
Wild animals	*Provide details on animals observed in or captured in the field; report species, sex and age where possible. Describe how animals were caught and transported and what happened to captive animals after the study (if killed, explain why and describe method; if released,*

say where and when) OR state that the study did not involve wild animals.

Field-collected samples	*For laboratory work with field-collected samples, describe all relevant parameters such as housing, maintenance, temperature, photoperiod and end-of-experiment protocol OR state that the study did not involve samples collected from the field.*
Ethics oversight	The protocols for animal maintenance and oocyte harvesting were approved by the Federation of European Laboratory Animal Science Associations (Berlin, Germany).

Note that full information on the approval of the study protocol must also be provided in the manuscript.
